# Sudden Visual Loss After Vitreoretinal Surgery: A Case Report

**DOI:** 10.1155/2024/6618094

**Published:** 2024-10-23

**Authors:** Agostino Salvatore Vaiano, Fabio Garavelli, Antonio Greco, Riccardo Merli, Alessandro De Filippis, Andrea Greco, Maria Marenco

**Affiliations:** Institute of Ophthalmology, Santa Croce e Carle Hospital, Cuneo, Italy

**Keywords:** case report, visual loss, vitreoretinal surgery

## Abstract

A 51-year-old male underwent vitrectomy with retrobulbar anesthesia for retinal detachment. Post surgery, he experienced systemic hypotension which normalized after 3 h. The day after, he complained of a central scotoma in the operated eye. Intraocular pressure was normal, but fundus examination revealed hemorrhages and whitening along the papillomacular bundle and macula, with additional whitening in the upper midperipheral region. Multimodal imaging confirmed branch retinal vein, artery, and cilioretinal artery occlusion. Further examination revealed mild-to-moderate obstructive sleep apnea syndrome. Vascular occlusions are potential complications of vitreoretinal surgery, warranting thorough preoperative assessment for underlying risk factors, even if causative mechanism is still unknown.

## 1. Introduction

Retinal vascular occlusions following anterior and posterior segment surgeries have been reported, with suggested risk factors [1]. Although rare, retinal vascular occlusions involving both veins and arteries, with various combinations, have been documented [2]. The central retinal artery supplies the retina primarily, while the cilioretinal artery, originating from a different vascular system, supplies a variable portion in certain eyes. The cilioretinal artery lacks autoregulation unlike the central retinal artery [3].

Here, we report a case of combination of cilioretinal artery occlusion (CLRAO) both with branch retinal vein occlusion (BRVO) and branch retinal artery occlusion (BRAO) soon after vitreoretinal surgery.

## 2. Case Presentation

A 51-year-old man engineer with a history of gastroesophageal reflux disease and refractive lens exchange in both eyes presented with flashes, floaters, and a curtain-like sensation in his left eye (LE) for 3 days. He had experienced three episodes of low blood pressure related to strong emotions over the past year.

**Figure 1:**
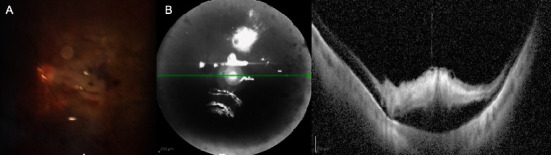
Left eye 4 days after surgery. (A) Slit lamp's fundus photo showing hemorrhages and whitening along the papillomacular bundle and in the macular region. (B) Optical coherence tomography showing hyperreflectivity of inner retinal layers in the macular area with increased central macular thickness.

Ophthalmic examination revealed best-corrected visual acuity (BCVA) of 20/20 in the right eye (RE) and 20/63 in the LE, with intraocular pressure (IOP) of 14 mmHg in both eyes. Fundus examination in the LE revealed a rhegmatogenous retinal detachment (RRD) in the superonasal quadrant with a horseshoe tear at 10 o'clock, sparing the macula. A vitrectomy under retrobulbar anesthesia was performed the following day. The retrobulbar nerve block involved injecting a mixture of ropivacaine, lidocaine, and hyaluronidase and was performed by an experienced anesthesiologist. The preservative-free solution, which did not contain adrenaline, was administered 30 min before the start of surgery. IOP was maintained below 20 mmHg throughout the procedure. Surgery involved core and base vitrectomy, perfluorocarbon liquid exchange, laser barrage, and 20% sulfur hexafluoride exchange for tamponade. The patient was instructed to remain in the supine position for the next 24 h. Immediately after surgery, the patient experienced systemic hypotension (blood pressure 89/52) and pressure normalized only after 3 h. The following day, despite the patient experiencing blurred vision due to the gas, he complained of the presence of a central scotoma in the LE, with hand motion BCVA and 13 mmHg IOP. Although the retina remained attached, flame hemorrhages and whitening were observed along the papillomacular bundle and in the macular region (Figure 1(A)), with retinal whitening in the superior midperiphery. Optical coherence tomography (OCT) showed inner retinal layer hyperreflectivity, suggesting macular ischemia (Figure 1(B)). Laboratory tests revealed positive IgG for HSV 1 and HZV, Factor II activity of 125.8 (normal range 70–120), and Factor V activity of 131.6 (normal range 50–120) (Table 1). Further cardiac and neurological investigations were normal. Polysomnography revealed mild-to-moderate obstructive sleep apnea syndrome (OSAS) with a clear postural impact.

**Figure 2:**
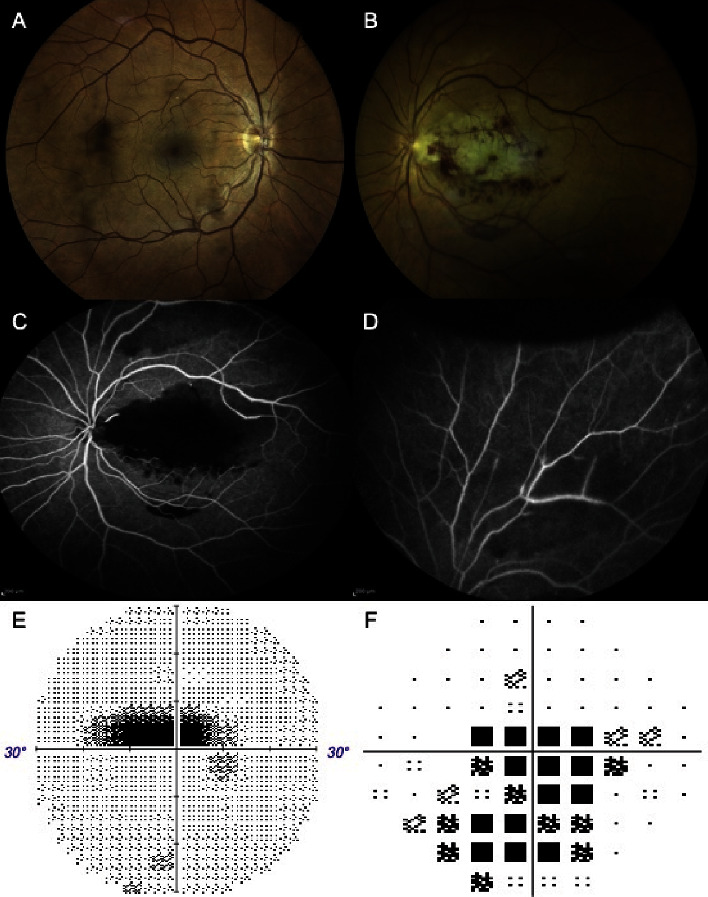
Fifteen days postoperatively. (A) Fundus photo of the right eye showing cilioretinal artery and (B) of the left eye revealing macular and papillomacular bundle whitening, superior midperipheral retinal whitening. (C, D) Fluorescein angiography indicating perfusion absence in multiple areas and obscured choroidal fluorescence. (E, F) Visual field indicating central scotoma and inferior altitudinal defect.

Fifteen days later, fundus photography revealed normal appearance in the RE with the presence of a cilioretinal artery (Figure 2(A)), while abnormalities persisted in the LE (Figure 2(B)). Fluorescein angiography (FA) demonstrated prolonged arteriovenous transit time, absence of perfusion in the territory of the cilioretinal artery and of some of the branches of the superior temporal arterial arcade, the masking of choroidal fluorescence by intraretinal hemorrhages along the papillomacular bundle and the macular region, and BRAO in the superior retinal midperiphery (Figures 2(C) and 2(D)). The visual field examination showed a central scotoma and an inferior altitudinal defect (Figures 2(E) and 2(F)).

**Figure 3:**
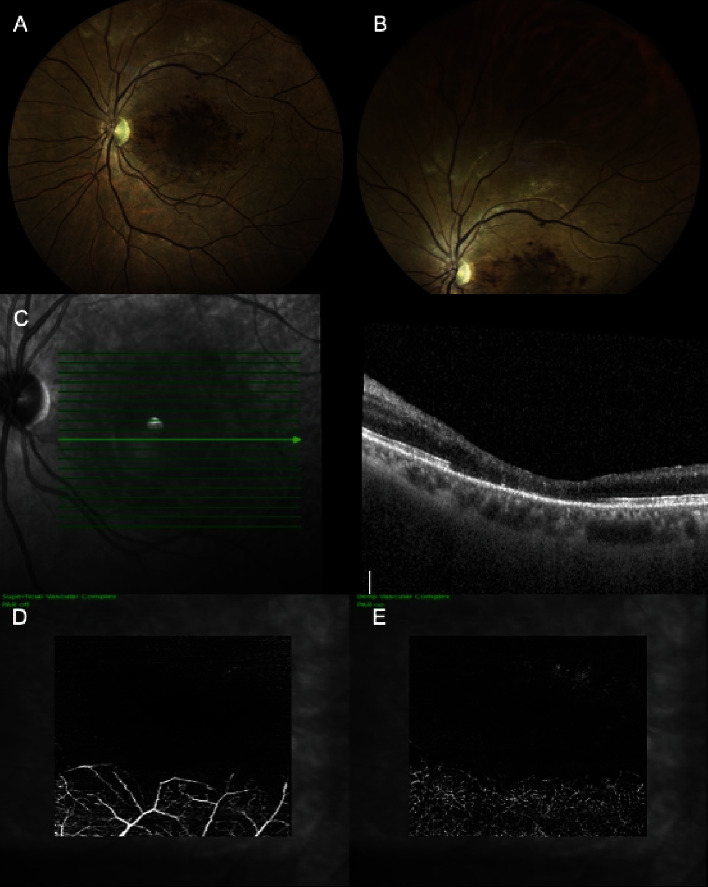
Left eye at 4-month follow-up. (A, B) Fundoscopy showing retinal hemorrhages and whitening along papillomacular bundle and macula, and whitening in the upper quadrant. (C) Optical coherence tomography revealing macular thinning. (D, E) Superficial and deep vascular complex in optical coherence tomography angiography showing the absence of perfusion in the macula and adjacent upper region.

After 4 months, the LE exhibited 20/63 with searching BCVA, 12 mmHg IOP, and persistent retinal abnormalities (Figures 3(A) and 3(B)). OCT showed macular atrophy (Figure 3(C)), and OCT-angiography (OCT-A) of both the superficial and deep vascular complexes revealed a lack of blood flow in both the macular area and surrounding upper region (Figures 3(D) and 3(E)).

## 3. Discussion

Retinal vascular occlusions can occur after vitreoretinal surgery, with potential risk factors including anesthesia, elevated IOP, perfusion issues, direct vessel injury, and ocular/systemic diseases [1].

To the best of our knowledge, retrobulbar anesthesia has been associated in the literature with retinal arterial occlusions or with central retina artery occlusion (CRAO) combined with central retinal vein occlusion (CRVO) [4]. In our case, occlusions such as CLRAO, BRVO, and BRAO were observed.

Cases of CRAO have been reported following retrobulbar anesthesia, with suggested causes including direct trauma to the artery or a pharmacological or compressive effect of the injected solution [5]. In our patient, it seems unlikely that the anesthesia could have caused a direct trauma or a compressive effect on the vessel because BRAOs were observed. Neither a pharmacological effect of the injected solution seems possible because it was based on preservative-free ropivacaine. In fact, cases of retinal artery occlusions were reported after uncomplicated vitreoretinal surgery following retrobulbar anesthesia with a preservative-containing mepivacaine formulation, suggesting a potential vasculotoxic effect of preservatives in mepivacaine formulations [5]. Moreover, hyaluronidase, when added to the local anesthetic, may offer partial protection against the reduction of pulsatile ocular blood flow and therefore lessening the risk of CRAO [4]. In our case, hyaluronidase was used as an adjuvant to ropivacaine and lidocaine. Furthermore, our anesthetic solution did not contain adrenaline, a substance that could have promoted the onset of arterial occlusion due to potential vasospasm [4].

Mild-to-moderate OSAS in our patient could have contributed to the vascular occlusions since they are included among the risk factors for venous occlusions [1, 6, 7].

Reduced ocular perfusion pressure (OPP) is strongly associated with retinal vein occlusions [1]. Sleep and supine positioning can lead to decreased blood pressure and OPP, potentially contributing to isolated CLRAO due to its lack of autoregulation [8].

CLRAO often co-occurs with CRVO, but cases of BRVO with CLRAO have been reported [2, 9]. Various hypotheses have been suggested to elucidate the occurrence of combined CRVO and CLRAO [2]. A sudden increase in intraluminal retinal capillary bed pressure secondary to CRVO could lead to CLRAO because reduced OPP due to CRVO may hinder cilioretinal artery adaptation [2]. Similarly, reduction in perfusion pressure of the cilioretinal and retinal arteries that may arise from a drop in systemic blood pressure, in turn, leads to decreased retinal circulation and subsequent venous stasis and thrombosis [2].

Episodes of systemic hypotension in our patient suggest abnormal autoregulation, potentially due to an abnormal vascular response to autonomic stimulation, local hormones, and metabolites [10]. In susceptible individuals, dysfunction in autoregulation permits OPP fluctuations (via changes in either systemic blood pressure or IOP) to alter retinal and optic nerve head perfusion [10–12]. Moreover, supine positioning can elevate IOP likely due to an elevation in episcleral venous pressure [11]. In our case, systemic hypotension following surgery and supine position could have reduced OPP leading to venous stasis causing BRVO followed by CLRAO. However, due to the insufficient information regarding whether manipulating OPP affects outcome, treatment aimed at increasing OPP (via means other than lowering IOP) is not justified at present [13]. Additionally, using drugs to lower IOP in the postoperative period could be beneficial.

To the best of our knowledge, this is the first case of a combination of BRVO, BRAO, and CLRAO following vitreoretinal surgery for RRD. Possible causes, potentially even interconnected, could have been reduced OPP, retrobulbar anesthesia, or OSAS.

## 4. Conclusion

In conclusion, vascular occlusions are potential complications of vitreoretinal surgery, but the exact pathogenesis remains unclear. A thorough medical history appears essential to identify high-risk patients and monitoring systemic blood pressure seems to be an important factor for prevention.

## Figures and Tables

**Table 1:** Screening laboratory tests performed.

**Laboratory test**
Complete blood count with differential
Glucose test
Lipid profile
ESR
CRP
Thrombophilia screening
PT
aPTT
Fibrinogen
Activated protein C resistance
Prothrombin G20210A (Factor II mutation)
Factor V Leiden mutation
Homocysteine
AT III
Protein C
Protein S
LA
Anti-beta-2-glycoprotein I antibodies
Anti-cardiolipin antibodies
Factors II, V, and VIII
Immunological screening
ENA
ANA
ANCA
Infectious screening
IgM and IgG for HSV 1-2, HZV, CMV, toxoplasma, and borrelia
TPHA-VDRL
QuantiFERON

Abbreviations: ANA = antinuclear antibodies; ANCA = antineutrophil cytoplasmic antibodies; aPTT = activated partial thromboplastin time; AT III = Antithrombin III; CMV = cytomegalovirus; CRP = C-reactive protein; ENA = extractable nuclear antigens antibodies; ESR = erythrocyte Sedimentation rate; HSV = herpes simplex virus; Ig G = immunoglobulin G; Ig M = immunoglobulin M; LA = lupus anticoagulant; PT = prothrombin time; TPHA-VDRL = Treponema Pallidum Hemagglutination and Venereal Disease Research Laboratory; VZV = herpes zoster virus.

## Data Availability

The data that support the findings of this study are available from the corresponding author upon reasonable request.
